# Signalling Pathways of Inflammation and Cancer in Human Mononuclear Cells: Effect of Nanoparticle Air Pollutants

**DOI:** 10.3390/cells13161367

**Published:** 2024-08-17

**Authors:** Agata Niechoda, Maciej Roslan, Katarzyna Milewska, Piotr Szoka, Katarzyna Maciorowska, Adam Holownia

**Affiliations:** Department of Pharmacology, Medical University of Bialystok, Mickiewicza 2c, 15-222 Bialystok, Poland; agata.niechoda@gmail.com (A.N.); roslanmaciek@gmail.com (M.R.); katarzyna.milewska@umb.edu.pl (K.M.); szoka.piotr@gmail.com (P.S.); katarzynamaciorowska12@gmail.com (K.M.)

**Keywords:** air pollution, cancer, histone H2A.X, inflammation, monocyte–macrophage transition, NF-κB, particulate matter

## Abstract

Fine inhalable particulate matter (PM) triggers an inflammatory response in the airways and activates mononuclear cells, mediators of tissue homeostasis, and tumour-promoting inflammation. We have assessed ex vivo responses of human monocytes and monocyte-derived macrophages to standardised air pollutants: carbon black, urban dust, and nanoparticulate carbon black, focusing on their pro-inflammatory and DNA-damaging properties. None of the PM (100 μg/mL/24 h) was significantly toxic to the cells, aside from inducing oxidative stress, fractional DNA damage, and inhibiting phagocytosis. TNFα was only slightly increased. PM nanoparticles increase the expression and activate DNA-damage–related histone H2A.X as well as pro-inflammatory NF-κB. We have shown that the urban dust stimulates the pathway of DNA damage/repair via the selective post-translational phosphorylation of H2A.X while nanoparticulate carbon black increases inflammation via activation of NF-κB. Moreover, the inflammatory response to lipopolysaccharide was significantly stronger in macrophages pre-exposed to urban dust or nanoparticulate carbon black. Our data show that airborne nanoparticles induce PM-specific, epigenetic alterations in the subsets of cultured mononuclear cells, which may be quantified using binary fluorescence scatterplots. Such changes intercede with inflammatory signalling and highlight important molecular and cell-specific epigenetic mechanisms of tumour-promoting inflammation.

## 1. Introduction

Urban air contamination and the gradual decline in air quality are constantly growing problems [1]. Biomonitoring of air pollution in cultured cells is not a routine practice because it is challenging to build a laboratory model of cumulative exposure [2,3]. Moreover, airborne PM is a mixture of particles with diverse physicochemical and biological properties. In this study, we have focused on the effect of the standardised urban dust (UD), nanoparticulate carbon black (NPCB), and carbon black (CB) on human monocytes (M) and monocyte-derived macrophages (MDM) ex vivo.

Fine inhalable PM triggers an inflammatory response in the airways, resulting in adverse health effects [4]. Respiratory system cancers are also associated with chronic inflammation. PM triggers oxidative stress and DNA damage, but the impact of PM on monocyte–macrophage transition is elusive [5,6]. In experimental studies, M is usually polarised to type 0 macrophages with a protein kinase C activator—phorbol 12-myristate 13-acetate (PMA). Polarised, macrophage-like cells have higher oxidative metabolism, increased phagocytosis, better proteolytic properties, altered pathogen-specific memory patterns, and differ in terms of kinase subsets and phosphorylation patterns [7,8]. M differentiation results in increased adherence, improved immune function, and loss of proliferation [8]. Alveolar macrophages are essential in PM-mediated inflammatory responses in the airways [9,10]. Along with epithelial cells, those are the initial cells encountering inhaled particles [11]. Their primary function is to phagocyte foreign particles but activated macrophages also produce reactive oxygen species (ROS) and release pro-inflammatory TNFα and IL-1 [12]. Naïve and glutathione-depleted THP1 cells grown with UD have shown a variety of activation patterns, diverse autophagy, and altered proteostasis, probably due to oxidative stress and protein damage [13]. It is evident that ROS and redox imbalance may modify signal transduction pathways crucial for autophagy and phagocytosis.

Now, we have combined the classical numerical flow cytometry and graphical binary fluorescence scatterplots to quantify and observe nanoparticle-induced epigenetic changes related to tumour-promoting inflammation. We have examined the effect of sub-cytotoxic concentrations of coarse CB, UD, and NPCB on naïve and phosphorylated nuclear factor kappa-light-chain-enhancer of activated B cells (NF-κB P-Ser 536) and naïve and phosphorylated histone H2A.X (γH2A.X) as markers of inflammation and DNA damage, respectively. We expect to show the mutual relationship between airway inflammation and lung cancer and highlight epigenetic molecular and cellular mechanisms of tumour-promoting inflammation induced by PM.

## 2. Materials and Methods

### 2.1. Cell Cultures, Monocyte–Macrophage Transition, and Treatment

The human primary M and a human monocyte–macrophage cell line (THP1 cells; ATCC^®^ TIB202™, Manassas, VA, USA) were used in this study. THP1 cells were used in all preliminary experiments due to the limited numbers of human M available. Peripheral blood mononuclear cells were isolated from the blood of healthy donors and were purified using CD14^+^ beads (Miltenyi Biotech, Bergisch Gladbach, Germany). Buffy coats were collected upon the approval of the Ethics Committee of the Medical University of Bialystok (decision #R-I-002/634/2018). M were grown in ATCC-formulated RPMI 1640 medium, supplemented with 2-mercapto-ethanol to a final concentration of 0.05 mM, 2 mM L-glutamine, 5% FCS, and PenStrep (100 U/mL). The cells were maintained at 37 °C in an incubator in a humidified atmosphere containing 5% CO_2_. For particular experiments, the cells were plated out onto 6-well (for flow cytometry) or 12-well (for ELISA or phagocytosis) plates and grown in control or PM-conditioned media for 24 h. M were differentiated into MDM, by adding 100 μg/mL PMA (Sigma-Aldrich, St. Louis, MI, USA) for 72 h at 37 °C, 5% CO_2_. Culture media supplemented with PMs were prepared using commercial, standardised urban dust (UD; Standard Reference Material 1649a the particle size 0.2–110 nm, with a mean size of about 10 nm), which was purchased from the National Institute of Standards and Technology (Gaithersburg, MD, USA), nanoparticulate carbon black (NPCB; 14 nm diameter, Printex 90; Degussa, Frankfurt, Germany), while coarse carbon black (CB; 260 nm diameter, Huber 990; Haeffner and Co., Ltd., Chepstow, UK) was used as a reference substance. The particles were suspended in a serum-free culture medium at a 100 µg/mL concentration and were sonicated in a Bandelin Sonoplus ultrasonic homogeniser for the 30 s before use. This concentration had only limited toxicity to THP1 cells (less than 10%), as evidenced in LDH release experiments. For experiments, THP1 cells and M were plated at a density of 0.15 × 10^6^ cells per well in Petri dishes and grown overnight to 80% confluency (THP1 cells). Then the medium was replaced with serum-free medium supplemented with CB, UD, or NPCB for a further 24 h. Cell-free controls were included in each experiment to assess the interference of particles with each assay. Positive controls were obtained with 2.5 μg/mL cisplatin (DNA damage), 50 μM tert butyl hydroperoxide (TBH; oxidative stress), or (100 ng/mL) lipopolysaccharide (LPS; from *E. coli*. O111:B4, Sigma-Aldrich L2630) applied to the cells for 24 h.

### 2.2. Quantification of Integrin—(CD11a/CD18), Macrophage Differentiation Marker—CD14 and Infiltration/Activation Marker—CD68

Cells were treated as described earlier, incubated in a blocking solution containing 5% normal mouse serum, 5% normal rat serum, and 1% FcBlock (eBiosciences, San Diego, CA, USA) in PBS and then stained with a standard panel of immunophenotyping antibodies against CD14, CD18, CD11a, and CD68 for 30 min at room temperature. After staining, cells were washed and fixed with 0.4% paraformaldehyde in PBS. Data were acquired with a Beckman Coulter CytoFlex flow cytometer (Beckman Coulter, Warsaw, Poland) using Kaluza Software (version 2.1.2).

### 2.3. Cytotoxicity

M or MDM were incubated with 25, 50, 100, or 200 μg/mL PM at 37 °C for 24 h. Cytotoxicity was determined by measuring the activity of lactate dehydrogenase (LDH) released into the supernatant using (The CyQUANT LDH Cytotoxicity Assay, Life Technologies Corporation, Eugene, OR, USA).

### 2.4. DNA Damage

**Figure 1:**
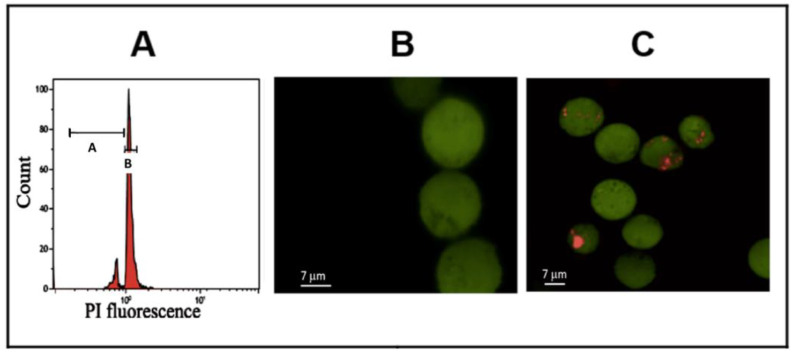
Panel (**A**) shows DNA histogram (propidium iodide fluorescence) with damaged (A) and undamaged (B) THP1 cells, (**B**)—micrographs of intracellular oxidative stress (DCF fluorescence), and (**C**) phagocytosis (phycoerythrin/DCF fluorescence). Micrographs were obtained with a Zeiss AXIO microscope (Zeiss Microscopy, Koln, Germany).

DNA damage was quantified in M and MDM by flow cytometry based on the quantity of the cellular DNA bound to fluorescent propidium iodide (PI). The distribution of red fluorescence was assessed on a Coulter CytoFlex flow cytometer (Beckman Coulter, Warsaw, Poland). Fractions of damaged and naïve M and MDM were quantified with DNA analysis software (MultiCycle, Phoenix Flow Systems Inc., San Diego, CA, USA, version AV-stand alone) according to their relative distribution in the sub-diploid (A) and diploid (B) fractions (Figure 1A).

### 2.5. Measurement of Intracellular Oxidative Stress

The intracellular generation of ROS was analysed using flow cytometry with carboxy-H2DCFDA a cell-permeant indicator (Figure 1B). Histograms of green fluorescence distribution were analysed in 10,000 cells on a Coulter CytoFlex flow cytometer (Beckman Coulter Life Sciences, Indianapolis, IN, USA). 

### 2.6. Phagocytosis Assays

M and MDM were treated with the PM as described earlier. Then, the cells were washed with washing buffer and incubated for two hours with latex beads (Cayman’s Phagocytosis Assay Kit, Cayman Chemical, Ann Arbor, MI, USA) coated with rabbit IgG labeled with FITC or PE (Figure 1C). The cells were washed twice with washing buffer and the engulfed fluorescent beads were detected using a Coulter CytoFlex flow cytometer (Beckman Coulter Life Sciences, Indianapolis, IN, USA).

### 2.7. TNFα Assay

TNFα was quantified in the culture medium of M and MDM with the Multi-Analyte Inflammatory Cytokine ELISArray Kit (Qiagen, Manchester, UK).

### 2.8. qRT Analyses: mRNA of H2AX and NF-κB

Total RNA was isolated using a RNeasy Micro Kit (Qiagen, Hilden, Germany). QRT-PCR was performed in a Stratagene Mx3005P QPCR System (Agilent Technologies, Santa Clara, CA, USA) with SG qPCR Master Mix (EURX, Gdansk, Poland) according to standard protocol. Primer sequences were: H2AX forward primer: GGCCTCCCAGGAGTACTAAGA, reverse primer: CTCTTTCCATGAGGGCGGTG; NF-κB forward primer GCAGCACTACTTCTTGACCACC, reverse primer TCTGCTCCTGAGCATTGACGTC; GAPDH forward primer: GATGGGTGGAGTCGCGT, reverse primer: CAGAGTTAAAAGCAGCCCTGG. The relative quantities were calculated against those of the *GAPDH* housekeeping gene using the ΔΔCt method including the PCR amplification efficiency of each gene.

### 2.9. Expression of NF-κB, NF-κB P-Ser 536, H2A.X, and γH2A.X

M and MDM were treated with the PM as described earlier. Cells were then fixed for 10 min in 4% methanol-free formaldehyde at room temperature and stained by direct labeling. NF-κB and H2A.X proteins were stained with specific rabbit fluorescent monoclonal antibodies (Cell Signalling, Danvers, MA, USA) recognising human NF-κB p65, phosphorylated NF-κB p56 (Ser 536) Alexa fluor 488 conjugate (green) or Alexa Fluor 647 conjugate (red) or human histone H2A.X and phosphorylated histone H2A.X (Ser 139) Alexa fluor 488 or Alexa Fluor 647 conjugates, and positive and negative controls. Antibodies were diluted in permeabilisation/wash buffer consisting of 0.1% Triton-X-100 (Sigma Chemical Company, Poznan, Poland) in phosphate-buffered saline with 1% bovine serum albumin. Samples were incubated for 20 min at room temperature in the dark, washed, filtered via a 100 μm cell strainer to remove aggregates, and analysed by Coulter CytoFlex flow cytometer (Beckman Coulter, Warsaw, Poland). The green fluorescence from Alexa Fluor 488 was excited using a 488 nm laser paired with a 530/30 nm bandpass filter, and the red fluorescence of the Alexa Fluor 647 was collected in the Cy5 channel. Doublet discrimination was performed manually by plotting the FSC signal area versus the FSC signal height on a linear scale. Colour compensation was set using unstained cells, single-stained cells, and double-stained cells. Positive controls including cells pretreated with LPS, TBH, or cisplatin were included as described earlier.

### 2.10. Binary Fluorescence Scatterplots of NF-κB, NF-κB P-Ser 536, H2A.X, and γ2A.X

Changes in NF-κB, NF-κB P-Ser 536, H2A.X, and γH2A.X expressions were assessed in double fluorescence experiments to delineate the relationship between both parameters and assess the response of particular cells. We addressed the issue by single and double staining M and MDM using epitope-specific rabbit monoclonal antibodies conjugated to Alexa Fluor 647 and Alexa Fluor 488 (both from Cell Signalling, Danvers, MA, USA) as described earlier. Samples were analysed in flow cytometry (Beckman Coulter CytoFlex flow cytometer). Experimental data were plotted as histograms, bivariate cytograms, and fluorescence density plots, and were analysed for the vector, central tendency, and spread using the FlowJo (V10.8.1, Ashland, OR, USA) and Flowing Software (v.2.5.1. freeware, Turku University, Finland).

### 2.11. Statistical Analysis

Nonparametric ANOVA approaches with Bonferroni or Dunnett post-tests of data were used for selected data pairs to evaluate statistical significance. All hypotheses were two-sided and assessed at a 5% level of significance.

## 3. Results

### 3.1. M-MDM Transition

**Figure 2:**
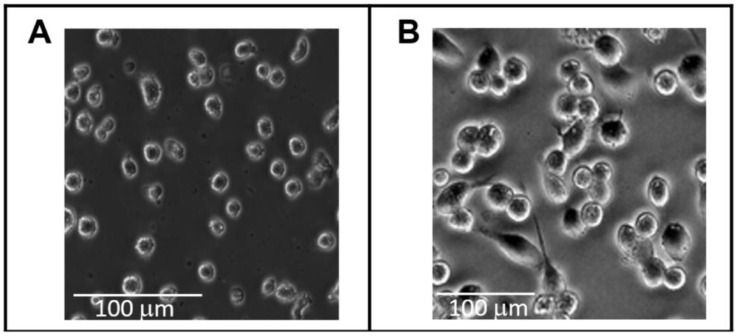
Phase contrast micrographs showing the morphology of (**A**) floating THP1 cells, and (**B**) adhering macrophage-like cells, differentiated in culture by phorbol-12-myristate 13-acetate. Micrographs were obtained with a Zeiss AXIO microscope.

The morphology of floating THP1 cells differentiated in culture by PMA into adhering macrophage-like cells is shown in Figure 2, panels A and B, respectively. M has been found to vividly react to PMA stimuli, leading to differentiation and polarisation into functionally, morphologically, and immunologically distinct cell types referred to as type 0 MDM.

Table 1 shows the effect of PMA on the expression of CD14, CA11a, CD18, and CD68 epitopes on human monocytes (M), and monocyte-derived macrophages (MDM). CD14 is a 55 kDa glycoprotein, a multifunctional LPS receptor that is constitutively expressed on the membrane of mature M and MDM. Almost four-fold higher expression of CD14 has been detected on MDM as compared to M. The CD11a and CD18 levels were also significantly higher (*p* < 0.01) in the MDM than in the M. Glycoprotein CD68 expression was increased more than twofold (*p* < 0.01) by cell transition.

### 3.2. Cytotoxicity, Oxidative Stress, Phagocytosis and Inflammation

#### 3.2.1. The Cell Membrane Damage Was Measured Using the Lactate Dehydrogenase (LDH) Release and Expressed in Terms of a Fraction of the Total Enzyme Activity

**Figure 3:**
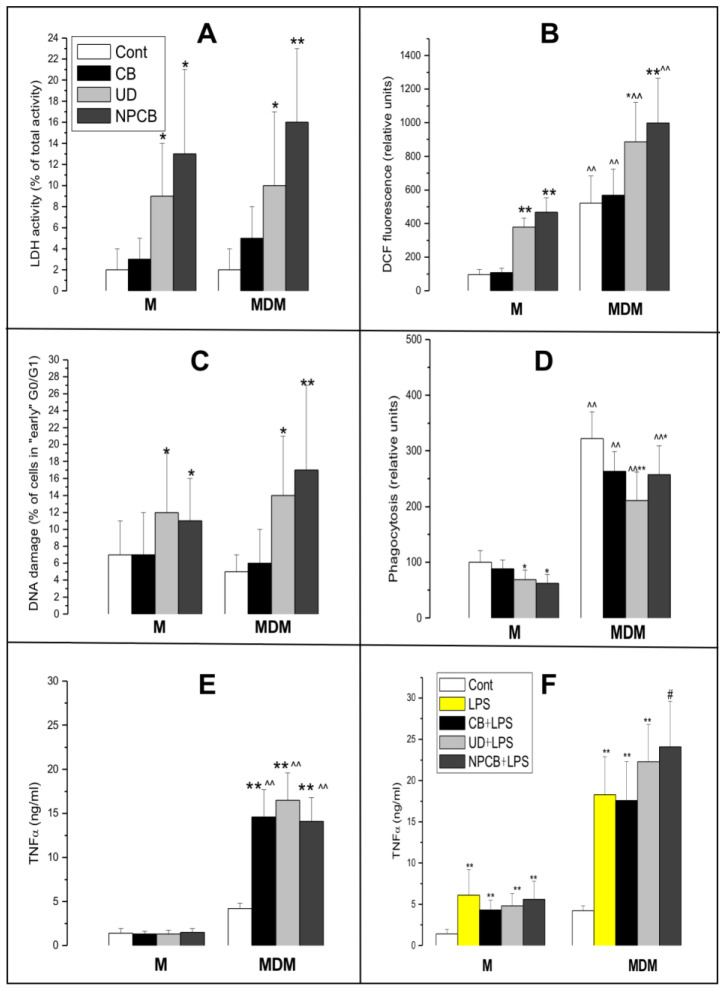
The effect of carbon black (CB), urban dust (UD), and nanoparticulate carbon black (NPCB) on cell membrane damage ((**A**); lactate dehydrogenase (LDH) release), oxidative stress (**B**), DNA damage (**C**), and phagocytosis (**D**) in human monocytes (M), and monocyte-derived macrophages (MDM). The inflammatory response (TNFα; (**E**)) to lipopolysaccharide (100 ng/mL for 24 h) is also included (**F**). All PMs were used at 100 µg/mL and the incubation time was 24 h. Cell transition was induced by phorbol-12-myristate 13-acetate (PMA; 100 nM) applied for 72 h. LDH was measured with CyQUANT LDH Cytotoxicity Assay kit, DNA ploidy was quantified using propidium iodide staining, intracellular oxidative stress was measured using 5-(and-6)-Carboxy-2′,7′-dichlorodihydrofluorescein diacetate (carboxy-H2DCFDA) while phagocytosis was assessed with fluorescent beads (Cayman Phagocytosis kit). Fluorescence was quantified with flow cytometry. TNFα was measured in the culture medium using a Multi-Analyte Inflammatory Cytokine ELISArray Kit (Quiagen, Wroclaw, Poland). Data are means ± SD of 6–10 assays. * *p* < 0.05; ** *p* < 0.01 for comparisons with the corresponding control cells; ^^ *p* < 0.01 for comparisons with corresponding naive cells; # *p* < 0.05 for comparisons with LPS-treated cells.

Table S1 and Figure 3 show the effect of PM on cell membrane damage (Figure 3A; LDH release), intracellular oxidative stress (Figure 3B), DNA damage (Figure 3C), phagocytosis (Figure 3D), and TNFα release from LPS-pretreated M (Figure 3E) and MDM (Figure 3F). The flow cytometry DNA histogram of PI fluorescence covering the zones of damaged (Figure 1A zone A) and euploid cells (Figure 1A zone B), intracellular oxidative stress (Figure 1B and phagocytosis Figure 1C are also shown. In the non-differentiated cells incubated with UD or NPCB, the fraction of cells with damaged membranes reached 9% (*p* < 0.05) and 13% (*p* < 0.05), respectively (Figure 3A). Similar values (10–16%) were obtained in the MDM (*p* < 0.01).

#### 3.2.2. Intracellular Oxidative Stress

Figure 3B increased after cell transition and PM treatment. First, in the MDM, the DCF fluorescence was about 5 times higher than in the naïve M cells (*p* < 0.01). In M, UD and NPCB but not CB increased the DCF fluorescence by 2.5 (*p* < 0.01) and 3.5 times (*p* < 0.01) and in MDM by elevated oxidative stress by about 1.5 times and 2 times (*p* < 0.05).

#### 3.2.3. Quantification of DNA Damage

Figure 3C revealed similar results, with approximately 12% DNA damage in the M treated with UD (*p* < 0.05) and 17% in those treated with NPCB (*p* < 0.01). In the MDM, the corresponding values were 14% (*p* < 0.05) and 17% (*p* < 0.01), respectively.

#### 3.2.4. Phagocytosis

Figure 3D significantly decreased in the M by approximately 31% (*p* < 0.05) when incubated with UD and by approximately 38% (*p* < 0.05) when treated with NPCB. In the MDM, the basic activity was about three times higher than in the M (*p* < 0.01). Both UD and NPCB decreased the phagocytosis in the MDM, respectively by 35% (*p* < 0.01) and 30% (*p* < 0.05).

#### 3.2.5. TNFα Was Unchanged in M Grown with PM

Figure 3E shows the transition of the M induced by PMA significantly increased inflammatory response, resulting in about a 3-fold increase in TNFα (*p* < 0.01) regardless of the kind of PM used. LPS used as a pro-inflammatory agent induced a strong (*p* < 0.01) pro-inflammatory reaction (Figure 3F). That effect was amplified by NPCB (*p* < 0.05) in MDM but not in M.

### 3.3. Expression and Activation of Histone H2A.X and NF-κB

**Figure 4:**
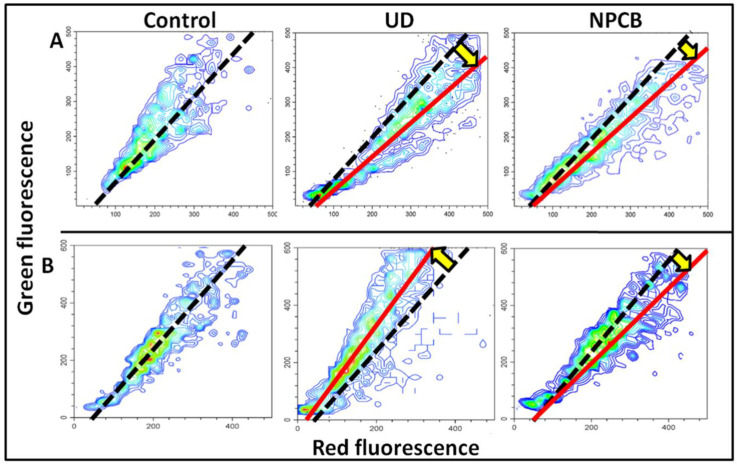
Representative flow cytometry scatterplots of double fluorescence (**A**)—green for naïve H2A.X protein, red for phosphorylated H2A.X, (**B**)—green for γH2A.X, red for NF-κB P-Ser 536 with different slopes of central tendency lines (dotted black lines). (**B**)-representative scatterplots of double fluorescence (green for γH2A.X, red for NF-κB P-Ser 536), with altered distribution of cells (vectors and spread) and opposite slopes of central tendency lines. Red lines are central tendency lines in cells treated with UD or NPCB, respectively.

The effect of CB, UD, and NPCB on histone H2A.X and nuclear factor kappa-light-chain-enhancer of activated B cells (NF-κB) mRNAs, proteins, and phosphorylated proteins is shown in Table 2, Figure S1A–D, and Figure 4. H2A.X is phosphorylated in response to DNA double-strand breaks (DSB), while NF-κB is a rapid-acting primary transcription factor that controls inflammation-related genes. In M, only UD increased (*p* < 0.05) γH2A.X protein (Figure S1A). Diverse (*p* < 0.05) slope values (Figure 4A) of γH2A.Xover H2A.X indicates that the phosphorylation of H2A.X is activated. NF-κB was activated in the M by both UD (*p* < 0.05) and slightly stronger (*p* < 0.01) by NPCB (Figure S1B). The naïve and phosphorylated forms of the protein increased by 37% (*p* < 0.05) and 43% (*p* < 0.05) as a result of the UD exposure, respectively, while the corresponding values for NPCB were 76% (*p* < 0.01) and 92% (*p* < 0.01), respectively. Binary scatterplots (Table 2, Figure 4B) of the naïve and phosphorylated proteins showed that the increase in the fraction of cells with phosphorylated NF-κB was more intense than the increase in the naïve proteins. In MDM, H2A.X partway was stimulated (*p* < 0.01) by UD but not by NPCB, while NF-κB was activated mostly by NPCB and less by UD (Table 2; Figure S1C,D).

## 4. Discussion

PM is the primary air pollutant in urban areas. In this study, we have aimed to compare and partly characterise the signalling pathways related to the inflammation and DNA damage of standardised nanoparticle air pollutants in human monocytes and macrophages. We have combined classical numerical flow cytometry and graphical binary fluorescence that preserve cell identity, to quantify and observe nanoparticle-induced epigenetic changes related to the tumour-promoting inflammation in the scatterplots of polarised cells. We have focused on the effect of the standardised UD, NPCB, and CB on human M and MDM ex vivo. The coarse CB is different from NPCB in terms of the primary diameter (260 nm vs. 14 nm), while UD is a complex, well-characterised, and natural but standardised substance that has many metal-bearing particles, polycyclic aromatic hydrocarbons, polychlorinated biphenyls, pesticides, and organic and inorganic constituents [14]. The reason why they have been used arises mainly from the defined physicochemical properties related to cytotoxicity and oxidative stress, i.e., the particle size, transition metals, or organic constituents activating specific membrane receptors.

Immune cells differentiate and polarise into functionally and immunologically varied cell types that are essential in airway inflammation. Typically, the monocytes treated with PMA differentiate into “M0” macrophages that are regarded as a transitory state. They may be further polarised into the M1 cells with IFN-γ and LPS or into the M2 phenotype with IL-4/IL-13. “M0” of MDM is closer to M1 than M2 [15]. The role of PM in the M differentiation and macrophage polarisation is still inconclusive [16]. PM2.5 has been indicated to increase the pro-inflammatory M1 polarisation via the “inorganic” ROS pathway, while the anti-inflammatory-M2 polarisation is “organic” and probably mTOR receptor-dependant [17]. Our experiments show that PM increases CD14 glycoprotein, a multifunctional lipopolysaccharide receptor in monocytes. Specifically, the highest increase was observed in the NPCB-treated M. The exposure to UD resulted in fewer significant changes, while CB even elevated CD14. Similar but fewer prominent changes were observed in the MDM, where the stimulating effect of CB was not detected. The expression of CD14 and CD16 has recently been used for differentiating between classical monocytes, and intermediate and non-classical monocytes [18], and this aspect merits further study. The CD11a levels in the naïve cells were not affected by PM but increased more than twofold in the polarised cells exposed to UD. Similar effects were observed in the CD18 expression. Both proteins play a major role in cell adhesion [19]. In fact, in our experiments, an increased monocyte adhesion after the PM exposure was noticed.

We have assessed the initial reactions of M and MDM to CB, UD, and NPCB in the pre-transitional and post-transition phases as well as the cell membrane damage. In both of the cell types, UD and NPCB produced low, about 8–13% cytotoxicity. This is significantly less than in the lung alveolar epithelial cells, where a similar PM burden generates significant cytotoxicity [20,21]. Our current data show that the cell membrane damage, DNA damage, and oxidative stress are similar at a relatively low cytotoxicity. A higher intracellular oxidative stress in the MDM is not associated with significantly higher DNA damage. Thus, macrophages may have efficient antioxidant and DNA-repairing systems, so oxidative stress is not the only factor causing PM-induced toxicity.

Alveolar macrophages decrease the inflammation in the respiratory tract by removing pathogens via phagocytosis. PM decreases macrophage phagocytosis in the experimental and clinical samples [22]. In the clinical samples, the carbon content in the airway macrophages has been proposed as a biomarker for PM exposure [23]. Both M and MDM recognise heterogeneous target particles either via pattern recognition receptors or opsonic receptors. In our experiments with monocytes, phagocytosis was reduced by UD and NPCB. In MDM, it was about three times higher than in the M but again both UD and NPCB reduced phagocytosis. A similar degree of inhibition of phagocytosis in both cell types by UD and NPCB indicates that the different chemical constitutions of PM may be irrelevant. In our experiments, phagocytosis in M was also reduced by UD and NPCB. In MDM, the basic activity was about three times higher than in the M, and again both UD and NPCB reduced phagocytosis. Disrupted phagocytosis may be related to the extracellular signal-regulated kinase 1/2 (ERK1/2) signalling, production of ROS, and deregulation of autophagy [24,25]. However, a similar degree of inhibition of phagocytosis in both cell types by UD and NPCB indicates that different chemical constitutions of PM and their specificity may not be relevant. On the other hand, the particle size may play a role, since large molecules of CB did not inhibit phagocytosis. Both phagocytosis and endocytosis depend on the phagocytic receptor engaged, but also on the 3D structure and physical properties of the particle [26,27]. Our data show that even phagocytosis of inorganic PM may contribute to pulmonary inflammation.

Only NPCB increased TNFα in MDM. An important observation is that when LPS stimulated the cells, both UD and NPCB-pretreated cells showed a significantly increased inflammatory response. TNFα gene does not require nucleosome remodelling [28].

LPS induces inflammatory responses primarily via the Toll-like receptor-TLR4-dependent NF-κB signalling pathway [28]. NF-κB is a “rapid-acting” primary transcription factor that controls many genes involved in inflammation [29,30]. We have shown that NF-κB expression is increased by UD and still more by NPCB. Both the naïve and phosphorylated forms of the protein increased in the effect of the NPCB and UD exposure. NF-κB regulates both the innate and adaptive immune responses [31,32,33]. NF-κB controls the transcription of genes that control inflammation, immune cell development, proliferation, phagocytosis, and cell death [33,34]. Phosphorylation of NF-κB at S536 leads to enhanced transactivation through the increased CBP/p300 binding [35]. However, NF-κB phosphorylation and its impact on transcriptional activity are complex due to many unspecific kinases and phosphorylation sites [36,37].

Histone phosphorylation is involved in the pathogenesis of inflammation, immune cell activation, proliferation, and apoptosis [38]. H2A.X phosphorylated at serine 139 is the first evidenced histone post-translational modification in response to DNA DSBs [38,39]. We have found that the H2A.X protein and γH2A.X are elevated in the subsets of the M and MDM exposed to UD. Moreover, the increase in the phosphorylated form has been found to be higher in the MDM and M than in the naïve protein. Phosphorylation of H2AX is required for the DNA repair and the activation of checkpoint proteins [40]. Various kinases may mediate the phosphorylation of H2A.X on serine 139, including ataxia-telangiectasia mutated (ATM), ATM and RAD3-related, and DNA-dependent protein kinase catalytic subunit. UD and NPCB-induced DSB may be, at least in part, repaired. As far as the binary scatterplots of NF-κB and H2A.X and the activated forms of both proteins are concerned, the cells indicated an intense polarisation and a tendency to scatter cells near the NF-κB axis for NPCB and the H2AX axis for UD. Both parameters yielded informative plots that reflected the intensity of stress, the degree of DNA damage, and the intensity of inflammation. The cells on the plots maintain their identity, and the plots may be further split and analysed by subpopulations with a high or low expression of both markers, which may facilitate biomonitoring.

## 5. Conclusions

In conclusion, we have partly characterised the molecular mechanisms linking inflammation, and the DNA damage caused by the NF-κBand histone H2A.X activation in M and MDM exposed to PM, and have complemented those findings with TNFα, DNA damage, oxidative stress, and phagocytosis. We have shown that PM with a variety of physical, chemical, and biochemical features share some common characteristics but substantially differ at the molecular level when defying immune cells. We postulate that the cell polarisation and the binary, numerical, and graphical analysis may be relatively convenient methods to study complex airborne compounds. In particular, we show that UD activates the histone H2A.X pathway, while NPCB predominantly triggers the NF-κB pathway, especially in macrophages. Moreover, we have evidenced that PM pre-exposure increases the inflammatory response of mononuclear cells to LPS. Our data also show that PM nanoparticles activate epigenetic alterations in the subsets of immune cells that intercede with inflammatory signalling and highlight important molecular and cellular mechanisms of tumour-promoting inflammation.

## Figures and Tables

**Table 1:** Changes in CD14, CA11a, CD18, and CD68 epitopes in human monocytes (M), and monocyte-derived macrophages (MDM). M to MDM transition was induced by phorbol-12-myristate 13-acetate (PMA; 100 nM; 72 h). Antigenic epitopes were quantified by flow cytometry using specific monoclonal, fluorescent antibodies.

	CD14	CD11a	CD18	CD68
M	100 ± 19	222 ± 43	122 ± 19	90 ± 24
MDM	398 ± 64 **	431 ± 75 **	435 ± 63 **	221 ± 34 **

** *p* < 0.01 for comparisons with non-polarised cells.

**Table 2:** The effect of carbon black (CB), urban dust (UD), and nanoparticulate carbon black (NPCB) on histone H2A.X, H2A.X phosphorylated at Ser 139 (γH2A.X) nuclear factor kappa-light-chain-enhancer of activated B cells (NF-κB) and NF-κB P-Ser 536, and their mRNAs. All units are relative. The scatter area and slopes of central tendency lines (a_ct_) of binary fluorescence scatterplots are included. All samples were run in triplicate and the experiment was repeated three times. Data are means ± SD.

M					
		**Control**	**CB**	**UD**	**NPCB**
H2A.X mRNA	Fold change	1.0 ± 0.22	1.15 ± 0.24	0.81 ± 0.27	0.98 ± 0.26
H2A.X protein	Relative expression	100 ± 21	108 ± 15	149 ± 37	122 ± 24
gH2A.X	Relative units	100 ± 24	111 ± 18	172 ± 31 **	131 ± 33
Binary scatterplots of gH2A.X/H2A.X	Area (relative units)	100 ± 19	91 ± 24	126 ± 16 *	88 ± 23
	Central tendency line (slope a_ct_)	1.0 ± 0.1	0.9 ± 0.1	0.7 ± 0.2 *	0.9 ± 0.2
NF-kB mRNA	Fold change	1.0 ± 0.33	0.78 ± 0.31	1.57 ± 0.45	1.71 ± 0.47
NFkB protein	Relative expression	100 ± 20	88 ± 21	137 ± 21 *	176 ± 34 **
NF-kB P-Ser536	Relative units	100 ± 21	99 ± 26	143 ± 28 *	192 ± 45 **
Binary scatterplots of NF-kB P-Ser536/NF-kB	Scatterplot area (relative units)	100 ± 16	102 ± 22	104 ± 15	111 ± 16
	Central tendency line (slope a_ct_)	1.0 ± 0.1	1.0 ± 0.2	0.8 ± 0.1 *	0.6 ± 0.2 **
Binary scatterplots of H2A.X/NF-kB	Scatterplot area (relative units)	100 ± 16	111 ± 20	121 ± 25	132 ± 31
	Central tendency line (slope a_ct_)	1.0 ± 0.1	1.0 ± 0.1	1.2 ± 0.1 *	0.7 ± 0.2 **##
Binary scatterplots of gH2A.X/NF-kBP-Ser536	Scatterplot area	100 ± 21	108 ± 16	109 ± 23	122 ± 19
	Central tendency line (slope a_ct_)	1.0 ± 0.1	0.9 ± 0.2	1.3 ± 0.2 *	0.7 ± 0.2 **##
MDM					
H2A.X mRNA	Fold change	1.0 ± 0.27	0.95 ± 0.28	1.40 ± 0.42	1.91 ± 0.44
H2A.X protein	Relative expression	100 ± 19	119 ± 16	164 ± 44 *	137 ± 42
gH2A.X	Relative units	100 ± 21	115 ± 19	194 ± 45 **	146 ± 32
Binary scatterplots of g-H2A.X/H2A.X	Scatterplot area (relative units)	100 ± 19	99 ± 24	135 ± 33	87± 21
	Central tendency line (slope a_ct_)	1.0 ± 0.1	0.9 ± 0.2	0.6 ± 0.3 *	0.8 ± 0.2
NF-kB mRNA	Fold change	1.0 ± 0.23	1.12 ± 0.31	1.85 ± 0.45	0.91 ± 0.44
NF-kB protein	Relative expression	100 ± 20	97 ± 26	139 ± 34	198 ± 55 **
NF-kB P-Ser536	Relative units	100 ± 23	113 ± 27	165 ± 48 *	218 ± 55 **
Binary scatterplots of NF-kB P-Ser536/NF-kB	Scatterplot area (relative units)	100 ± 23	101 ± 19	105 ± 26	121 ± 25
	Central tendency line (slope a_ct_)	1 ± 0.1	1.3 ± 0.2 *	0.7 ± 0.1 **	0.5 ± 0.2 **
Binary scatterplots of H2A.X/NF-kB	Scatterplot area (relative units)	100 ± 19	121 ± 22	132 ± 23	133 ± 31
	Central tendency line (slope a_ct_)	1.0 ± 0.2	1.1 ± 0.2	1.3 ± 0.2 *	0.7 ± 0.3 **##
Binary scatterplots ofgH2A.X/NF-kBP-Ser53	Scatterplot area (relative units)	100 ± 15	122 ± 16	111 ± 25	125 ± 17 *
	Central tendency line (slope a_ct_)	1.1 ± 0.2	1.1 ± 0.2	1.5 ± 0.3 **	0.6 ± 0.3 **##

* *p* < 0.05; ** *p* < 0.01 for comparisons with the corresponding control cells. ## *p* < 0.01 for comparisons with UD-treated cells.

## Data Availability

The data presented in this study are available on request from the corresponding author due to privacy.

## Supplementary Materials

The following supporting information can be downloaded at: https://www.mdpi.com/article/10.3390/cells13161367/s1. Figure S1: The effect of carbon black (CB), urban dust (UD), and nanoparticulate carbon black (NPCB) on the expression of (A) histone H2A.X mRNA, protein, and H2A.X phosphorylated at Ser 139 (γH2A.X), nuclear factor kappa-light-chain-enhancer of activated B cells (NF-κB) mRNA, NF-κB protein and NF-κB protein phosphorylated at Ser 536 (NF-κB P-Ser 536) in monocytes (M; A and B), monocyte-derived macrophages (MDM; C and D). * *p* < 0.05; ** *p* < 0.01 for comparisons with the corresponding control cells. Table S1: The effect of carbon black (CB), urban dust (UD), and nanoparticulate carbon black (NPCB) on cell membrane damage (lactate dehydrogenase (LDH) release), cytotoxicity, proliferation, DNA damage, oxidative stress and phagocytosis in human monocytes (M), and monocyte-derived macrophages (MDM). The inflammatory response (TNFα to lipopolysaccharide (100 ng/mL for 24 h) is also included. All PM was used at 100 µg/mL and the incubation time was 24 h. Cell transition was induced by phorbol-12-myristate 13-acetate (PMA; 100 nM) applied for 72 h. LDH was measured with CyQUANT LDH Cytotoxicity Assay kit and DNA ploidy was quantified using propidium iodide staining, intracellular oxidative stress was measured using 5-(and-6)-Carboxy-2′,7′-dichlorodihydrofluorescein diacetate (carboxy-H2DCFDA) while phagocytosis was assessed with fluorescent beads (Cayman Phagocytosis kit). Fluorescence was quantified with flow cytometry. TNFα was measured in the culture medium using a Multi-Analyte Inflammatory Cytokine ELISArray Kit (Quiagen, Wroclaw, Poland). Data are means ± SD.
