# Supplementary material for: Signalling Pathways of Inflammation and Cancer in Human Mononuclear Cells: Effect of Nanoparticle Air Pollutants
Source: Cells. 2024 Aug 17;13(16):1367. doi: 10.3390/cells13161367 (PMC11352816; doi:10.3390/cells13161367)
Supplement: Supplementary file 1 [file cells-13-01367-s001.zip › Figure S1.pdf]

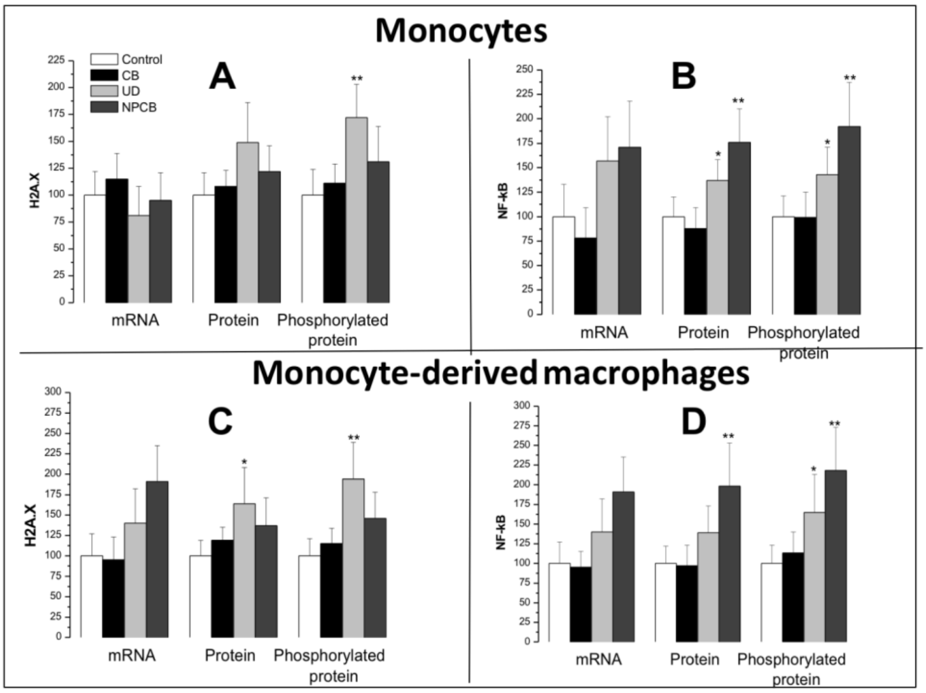

Figure S1. The effect of carbon black (CB), urban dust (UD), and nanoparticulate carbon black (NPCB) on the expression of (A) histone H2A.X mRNA, protein, and H2A.X phosphorylated at Ser 139 (γH2A.X), nuclear factor kappa-light-chain-enhancer of activated B cells (NF-κB) mRNA, NF-κB protein and NF-κB protein phosphorylated at Ser 536 (NF-κB P-Ser 536) in monocytes (M; A and B), monocyte-derived macrophages (MDM; C and D). *P<0.05; ** P<0.01 for comparisons with the corresponding control cells
