# Supplementary material for: Signalling Pathways of Inflammation and Cancer in Human Mononuclear Cells: Effect of Nanoparticle Air Pollutants
Source: Cells. 2024 Aug 17;13(16):1367. doi: 10.3390/cells13161367 (PMC11352816; doi:10.3390/cells13161367)
Supplement: Supplementary file 1 [file cells-13-01367-s001.zip › Table S1.pdf]

Table S1. The effect of carbon black (CB), urban dust (UD), and nanoparticulate carbon black (NPCB) on cell membrane damage (lactate dehydrogenase (LDH) release), cytotoxicity, proliferation, DNA damage, oxidative stress and phagocytosis in human monocytes (M), and monocyte-derived macrophages (MDM). The inflammatory response (TNFα) to lipopolysaccharide (100 ng/ml for 24 hours) is also included. All PM was used at 100 μg/mL and the incubation time was 24 hours. Cell transition was induced by phorbol-12-myristate 13-acetate (PMA; 100 nM) applied for 72 hours. LDH was measured with CyQUANT LDH Cytotoxicity Assay kit and DNA ploidy was quantified using propidium iodide staining, intracellular oxidative stress was measured using 5-(and-6)-Carboxy-2′,7′-dichlorodihydrofluorescein diacetate (carboxy-H2DCFDA) while phagocytosis was assessed with fluorescent beads (Cayman Phagocytosis kit). Fluorescence was quantified with flow cytometry. TNFα was measured in the culture medium using a Multi-Analyte Inflammatory Cytokine ELISArray Kit (Quiagen, Wroclaw, Poland). Data are means ± SD.

Cytotoxicity, oxidative stress, phagocytosis					
		Control	CB	UD	NPCB
LDH release (% of total activity)					
	M	3 ± 2	3 ± 2	9 ± 5*	13 ± 8*
	MDM	2 ± 2	5 ± 3	10 ± 7*	16 ± 7**
DNA damage (%)					
Damaged DNA	M	7 ± 4	7 ± 5	12 ± 7*	11 ± 5*
	MDM	5 ± 2	6 ± 4	14 ± 7*	17 ± 10**
Haploid DNA	M	94 ± 8	93 ± 9	88 ± 9	89 ± 11
	MDM	95 ± 7	94 ± 11	86 ± 11	83 ± 13
Oxidative stress (relative units)					
	M	100 ± 31	108 ± 27	378 ± 55**	467 ± 87**
	MDM	322 ± 48^^	263 ± 36	211 ± 51**^^	257 ± 35 *^^
Phagocytosis (relative units)					
	M	100 ± 21	88 ± 16	69 ± 17*	62 ± 16*
	MDM	322 ± 48^^	263 ± 36	211 ± 51**^^	257 ± 35 *^^
TNFα (ng/ml)					
	M	1.4 ± 0.54	1.3 ± 0.31	1.3 ± 0.44	1.5 ± 0.41
	MDM	4.2 ± 0.6^^	14.6 ± 3.1	16.5 ± 2.8	14.1 ± 2.7
PMs+LPS Post-treatment	M	6.1.5 ± 3.1^^	4.3 ± 1.2	4.8 ± 1.5^^	5.6 ± 2.2**^^
	MDM	18.3 ± 4.6^^	17.6 ± 4.7	22.3 ± 4.5	24.1 ± 5.5#

*P<0.05;** P<0.01 for comparisons with the corresponding control cells

^ P<0.05; ^^ P<0.01 for comparisons with corresponding naive cells

# P<0.05; ## P<0.01 for comparisons with LPS-treated cells
